# Occurrence and antimicrobial resistance of *Salmonella* isolated from retail meats in Anhui, China

**DOI:** 10.1002/fsn3.2266

**Published:** 2021-07-16

**Authors:** Wu Wang, Jing Chen, Xuefei Shao, Pan Huang, Jing Zha, Yingwang Ye

**Affiliations:** ^1^ Engineering Research Center of Bio‐process Ministry of Education Hefei University of Technology Hefei China; ^2^ School of Food and Biological Engineering Hefei University of Technology Hefei China

**Keywords:** antimicrobial resistance, resistance genes, retail meat, *Salmonella*, virulence genes

## Abstract

*Salmonella* is considered one of the major foodborne pathogens associated with severe infections. Little attempt has been focused on the distribution of *Salmonella* in retail meats and the analysis of its phenotypic characteristics in Anhui Province. The aim of this study was to characterize the prevalence of *Salmonella* serovars, antimicrobial susceptibility, antimicrobial resistance genes, and virulence genes in *Salmonella* recovered from retail meats in Anhui, China. Out of the 120 samples collected from supermarket chains and open‐air markets, 16 samples (13.3%) were positive for *Salmonella*, of which *Salmonella enterica* serovars Enteritidis and Typhimurium were the common serotypes. Significant differences in incidence were found between supermarket chains and open‐air markets (*p* < 0.05). Overall, all 16 isolates were resistant to at least two tested antimicrobials, while 12 isolates showed multiple antimicrobial resistant phenotypes. High resistance was observed for ampicillin (87.5%), doxycycline (75.0%), and tetracycline (62.5%). The *sul2* was detected in all isolates, and the *aac(6′)‐Ib‐cr* (93.8%) and the *tetA* (81.3%) were predominant in 10 resistance genes conferring five classes of antimicrobials. In addition, the correlation between resistance phenotypes and genes of tetracyclines and aminoglycosides was more than 80%. Interestingly, all the *Salmonella* isolates contained the genes *mogA*, *mgtC*, *sopB*, and *spvB*, whereas the *siiE* was variably represented. The findings in this study showed high prevalence, antimicrobial resistance, and the existence of virulence genes, suggesting that effective measures are required to ensure microbial safety from retail meats.

## Graphical abstract

The study was to investigate the prevalence of *Salmonella* serovars, antimicrobial susceptibility, antimicrobial resistance genes and virulence genes in *Salmonella* recovered from retail meats in Anhui to analyze the relationship between antimicrobial resistance genes and antimicrobial susceptibility. The findings in this study showed high prevalence, antimicrobial resistance, and the existence of virulence genes, suggesting that effective measures are required to ensure microbial safety from retail meats.

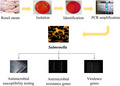

## 1 INTRODUCTION

*Salmonella* is the most common and representative pathogen isolated from confirmed cases of global foodborne diseases, which poses serious risks to human health (Cunha‐Neto et al., 2017). In most cases, the transmission to humans is associated with the consumption of contaminated foods or water, the treatment of contaminated raw meat, cross‐contamination, and the consumption of uncooked foods containing *Salmonella* (Yang et al., 2014). *Salmonella* is considered the main cause of outbreaks of foodborne infections reported in the European Union, and the number of cases has not shown any statistically significant decrease over the past 5 years from 2013 to 2017 (EFSA, 2018). An estimated 23% of bacterial foodborne poisoning is caused by *Salmonella* in Korea (Kim et al., 2016). Similarly in China, *Salmonella* is responsible for 19% of foodborne illnesses (Li et al., 2020).

Meats are likely to be contaminated with *Salmonella* at any stage during the slaughtering process and cross‐contaminated during subsequent processing, distribution, marketing, and handling (Jiang et al., 2021), that is why meat samples are more frequently involved in food infection cases caused by *Salmonella*. Therefore, the detection of *Salmonella* in retail meats is an indispensable part of food microbial inspection. In France, undercooked beef accounts for 35% of foodborne *Salmonella* infections, and consumption of contaminated poultry products is also a significant food safety risk in the USA (Velasquez et al., 2018). Mathole et al. (2017) indicated that 19% of poultry were infected with *Salmonella* in South Africa. Based on the surveillance of *Salmonella* contamination on raw poultry in 20 provinces of China, it is worth noting that the detection rate of *Salmonella* was 15.83% in retail markets (Li, Pei, et al., 2019). Despite this, however, attention on *Salmonella* is not limited to its global existence, but the major focus of food safety is the emergence of multiple antimicrobial resistant (MDR) *Salmonella*.

Recently, the widespread overuse and abuse of antimicrobial agents in food animal production and human medicine have contributed to the increasing incidence of antimicrobial resistance, particularly MDR (Arslan & Eyi, 2010). Antimicrobial resistance is recognized as one of the major challenges of public health worldwide in the twenty‐first century (Doménech et al., 2015). Notably, according to available data, China experienced the fastest growth rate worldwide in antimicrobial resistance of pathogens (Ren et al., 2016). With the widespread use of antimicrobials in livestock production, resistant isolates of *Salmonella* and their antimicrobial resistance can spread quickly to the human population by the consumption of products from contaminated meat (Zhu et al., 2019). The intensity of resistance varies globally, but the resistance rate is on the rise (Ifeanyichukwu et al., 2017; Louden et al., 2012; Mąka et al., 2015; Velasquez et al., 2018). To better understand the resistance mechanisms, it is necessary to detect antimicrobial resistance genes in *Salmonella* isolated from retail meats. In particular, genes active against β‐lactams and fluoroquinolones, which are first‐line antimicrobials, have been analyzed.

The pathogenicity of *Salmonella* is mediated by the interaction of numerous virulence genes, located in the *Salmonella* pathogenicity islands (SPIs), plasmids, lipopolysaccharides, and enterotoxins (Farahani et al., 2018). SPIs are clusters of virulence genes on chromosomes containing most of the virulence genes of *Salmonella* (Chaudhary et al., 2015). To date, 23 SPIs have been identified in *Salmonella*, of which SPI‐1 to SPI‐5 have been well studied (Ben Salem et al., 2017). In addition, plasmid‐borne virulence genes, such as *spvB* and *spvC*, have been reported to promote the survival and growth of *Salmonella* in host cells, aggravating the severity of enteritis (Oueslati et al., 2016). Although the prevalence of *Salmonella* in retail meats in China has been reported, there are few studies on virulence genes carried by *Salmonella*.

China has a high incidence of salmonellosis, with animal‐derived foods recognized as the major reservoirs of *Salmonella* dissemination, especially chicken, pork, and duck (Yang et al., 2019). Most investigations of prevalence and resistance of *Salmonella* in Anhui Province, a major province of livestock production and consumption in China, have focused on clinical and veterinary strains (Wang et al., 2013). However, little information on the incidence and antimicrobial resistant of *Salmonella* in retail meats exposed to consumers in Anhui is available, and there is a lack of epidemiological data for risk assessment. The study targeted chicken, pork, and duck that are collected by supermarket chains and open‐air markets.

The purpose of the present study was to investigate the prevalence of *Salmonella* and to further examine antimicrobial resistance, antimicrobial resistance genes, and virulence genes of *Salmonella* isolated from retail meats in Anhui, China.

## 2 MATERIALS AND METHODS

### 2.1 Sample collection

A total of 120 retail meat samples, including pork (*n* = 45), chicken (*n* = 45), and duck (*n* = 30), were collected randomly from different supermarket chains and open‐air markets in Hefei, Anhui Province. Immediately following purchase, the samples were aseptically packed into polyethylene bags containing ice and transferred to the laboratory for testing within 3 h.

### 2.2 Isolation and identification of isolates based on *invA* sequencing

From each meat sample, *Salmonella* isolates were searched and purified according to methods described by Yang et al. (2016). *Salmonella* genomic DNA was extracted using a Bacterial DNA Extraction kit (Tiangen, Beijing, China) and stored at −20°C until further use. Isolates with typical *Salmonella* phenotypes were further identified using polymerase chain reaction (PCR) amplification of the invasion gene *invA* (Bülte & Jakob, 1995) synthesized by GENEWIZ. The concentrations of the amplification reagents and the PCR cycling conditions are described in section 2.4.

### 2.3 Antimicrobial susceptibility testing

Antimicrobial susceptibility testing was determined using the broth microdilution method in accordance with the recommendations of the Clinical and Laboratory Standards Institute (CLSI) (CLSI, 2017). Eleven antimicrobial agents belonging to six different antimicrobial classes were applied: ampicillin, ceftiofur, ciprofloxacin, doxycycline, enrofloxacin, florfenicol, gentamicin, ofloxacin, spectinomycin, sulfisoxazole, and tetracycline. For the various types of antimicrobials, the isolates were classified as sensitive (S), intermediate (I), or resistant (R) according to the CLSI guidelines (CLSI, 2017). The multiple antimicrobial resistance (MAR) index was determined as described previously (Krumperman, 1983). *Escherichia coli* ATCC 25922 was used as a quality control strain.

### 2.4 Detection of antimicrobial resistance and virulence genes

Collected *Salmonella* isolates were tested for the presence of resistance and virulence genes using PCR. The following genes associated with antimicrobial resistance for fluoroquinolones (*qnrS*, *aac(6′)‐Ib‐cr*), aminoglycosides (*aadA1*, *aadA2*), tetracyclines (*tetA*, *tetG*), sulfonamides (*sul1*, *sul2*), and β‐lactams (*bla*_TEM_, *bla*_CMY_) were detected. Furthermore, seven genes that contribute to virulence were screened. Two target genes (*spvB* and *spvC*) were located on virulence plasmids, and five (*mogA*, *sseL*, *mgtC*, *siiE*, *and sopB*) were located on SPI‐1 to SPI‐5. All the primer sequences and predicted sizes of the amplified products are listed in Table S1.

The PCR protocol for amplification was previously described by Hai et al. (2020). PCR was performed in a final volume of 25 μl consisting of 12.5 μl DreamTaq Green PCR Master Mix, 1 μl each of forward and reverse primers (0.4 μM), 2 μl template DNA, and 8.5 μl dH_2_O. After completion of the PCR reaction cycles, the products were run on 2.0% agarose gel for 30 min at 110 V using electrophoresis. For confirmation of the amplified genes, all of the PCR products were sequenced by Sangon Biotech Co., Ltd (Shanghai, China) using an ABI3730 sequencer. The sequences obtained were compared with the previously published genes in GenBank (https://www.ncbi.nlm.nih.gov/genbank/) using BLAST (https://blast.ncbi.nlm.nih.gov/Blast.cgi).

## 3 RESULTS

### 3.1 Prevalence of *Salmonella* in retail meats in Anhui

A total of 16 (13.3%) *Salmonella* isolates were identified from 120 retail meats of different animal origin analyzed in Anhui (Table 1). All the 16 isolates were positive for PCR based on *invA* sequencing. Of these isolates, 4 (14.3%) and 2 (9.5%) samples were obtained from chicken and duck samples in supermarket chains, respectively; 3 (13.6%), 6 (35.3%), and 1 (11.1%) were obtained from pork, chicken, and duck samples in open‐air markets, respectively. Overall, the prevalence of *Salmonella* in retail meats obtained from open‐air markets was significantly higher (*p* < 0.05) than that in supermarket chains.

**TABLE 1:** Distribution of *Salmonella* obtained from supermarket chains and open‐air markets in different retail meats

Location	Pork (%)	Chicken (%)	Duck (%)	Total (%)
Supermarket chain	0.0^a^ (0/23)	14.3^a^ (4/28)	9.5^a^ (2/21)	8.3^b^ (6/72)
Open‐air market	13.6^a^ (3/22)	35.3^a^ (6/17)	11.1^a^ (1/9)	20.8^a^ (10/48)
Total	6.7 (3/45)	22.2 (10/45)	10.0 (3/30)	13.3 (16/120)

Note

Values with different superscript letters (a‐b) indicate statistical significant differences between supermarket chains and open‐air markets (*p* < 0 .05).

Compared to the prevalence in pork (6.7%) and duck samples (10.0%), the prevalence of *Salmonella* was the highest in chicken (22.2%) among the retail meats tested. No *Salmonella* isolates were detected in the pork samples from supermarket chains. Further analysis revealed no significant difference (*p* > 0.05) in the overall contamination of *Salmonella* isolated from different retail meats.

The 16 isolates tested belonged to six serovars (Table 2). *Salmonella enterica* serovars Enteritidis and Typhimurium, both accounting for 31.3%, were the most frequently detected serotypes in the present study. The other four unusual serovars identified were *S*. Typhi, *S*. Paratyphi, *S*. Ouakam, and *S*. Goldcoast with an incidence of 18.8%, 6.3%, 6.3%, and 6.3%, respectively. It should be noted that *S*. Typhimurium was present in all three retail meats, while *S*. Enteritidis, *S*. Paratyphi, and *S*. Ouakam were found only in chicken.

**TABLE 2:** *Salmonella* serotypes collected from pork, chicken, and duck samples

Serotype	Pork	Chicken	Duck	No. of isolates (%)
*Salmonella* Enteritidis	0	5	0	31.3 (5/16)
*Salmonella* Typhimurium	2	1	2	31.3 (5/16)
*Salmonella* Typhi	0	2	1	18.8 (3/16)
*Salmonella* Goldcoast	1	0	0	6.3 (1/16)
*Salmonella* Ouakam	0	1	0	6.3 (1/16)
*Salmonella* Paratyphi	0	1	0	6.3 (1/16)

### 3.2 Antimicrobial resistance

Antimicrobial susceptibility tests of the 16 *Salmonella* isolates against 11 antimicrobial agents are presented in Table 3. The prevalence of resistance was 87.5% for ampicillin, 75.0% for doxycycline, 62.5% for tetracycline, 50.0% for sulfisoxazole, and 50.0% for florfenicol. For spectinomycin, the percentage of *Salmonella* resistance was 43.8%, followed by gentamicin, 25.0%. In comparison, enrofloxacin resistance (18.8%) was observed less frequently, and most of the isolates were sensitive to ciprofloxacin (87.5%). Only one isolate was resistant to ceftiofur (6.3%) and ofloxacin (6.3%). These results indicate that high resistance rates were detected against ampicillin, oxytetracycline, and tetracycline. Except for enrofloxacin and sulfisoxazole, there was no significant difference (*p* > 0.05) between resistance phenotypes and sampling place. Additionally, the results showed complete resistance to doxycycline from supermarket chains (100.0%) and ampicillin from open‐air markets (100.0%).

**TABLE 3:** Antimicrobial resistance of *Salmonella* among different supermarket chains and open‐air markets

Antimicrobial class	Antimicrobial agent	No. of resistant isolates (%)		
		Overall	Supermarket chain	Open‐air market
Tetracyclines	DOX	75.0 (12/16)	100.0 (6/6)	60.0 (6/10)
	TET	62.5 (10/16)	66.7 (4/6)	60.0 (6/10)
β‐Lactams	AMP	87.5 (14/16)	66.7 (4/6)	100.0 (10/10)
	CEF	6.3 (1/16)	16.7 (1/6)	0.0 (0/10)
Aminoglycosides	GEN	25.0 (4/16)	33.3 (2/6)	20.0 (2/10)
	SPT	43.8 (7/16)	33.3 (2/6)	50.0 (5/10)
Fluoroquinolones	CIP	12.5 (2/16)	16.7 (1/6)	10.0 (1/10)
	ENR	18.8 (3/16)	50.0 (3/6)	0.0 (0/10)
	OFL	6.3 (1/16)	16.7 (1/6)	0.0 (0/10)
Amphenicols	FFC	50.0 (8/16)	50.0 (3/6)	50.0 (5/10)
Sulfonamides	SUL	50.0 (8/16)	0.0 (0/6)	80.0 (8/10)

Abbreviations: AMP, ampicillin; CEF, ceftiofur; CIP, ciprofloxacin; DOX, doxycycline; ENR, enrofloxacin; FFC, florfenicol; GEN, gentamicin; OFL, ofloxacin; SPT, spectinomycin; SUL, sulfisoxazole; TET, tetracycline.

Table 4 shows 11 different patterns of antimicrobial resistance in *Salmonella*. Among the multiple resistance patterns displayed, resistance to doxycycline, tetracycline, ampicillin, and spectinomycin simultaneously existed in 43.8% (7/16) of the isolates. Ampicillin (87.5%) was present in almost all the multi‐resistance patterns. A single isolate of *S*. Goldcoast from pork was observed to display resistance to seven antimicrobial agents with antimicrobial resistance phenotype of DOX + TET + AMP + SPT + CIP + FFC + SUL and had the highest MAR index of 0.64. The other one and three isolates were resistant to seven and six antimicrobial agents with MAR indices of 0.64 and 0.55, respectively. Therefore, it could be concluded that MDR was increasingly serious in the food hygiene environment of open‐air markets.

**TABLE 4:** Antimicrobial resistance phenotype patterns and MAR indices of *Salmonella* from this study

Antimicrobial resistance phenotype pattern	No. of antimicrobials	No. of isolates	MAR index
DOX + AMP	2	1	0.18
AMP + SUL	2	3	0.18
AMP + FFC + SUL	3	1	0.27
DOX + TET + GEN + FFC	4	2	0.36
DOX + TET + AMP + SUL	4	1	0.36
DOX + TET + AMP + SPT + ENR	5	2	0.45
DOX + TET + AMP + SPT + SUL	5	1	0.45
DOX + TET + AMP + GEN + SPT + FFC	6	2	0.55
DOX + TET + AMP + SPT + FFC + SUL	6	1	0.55
DOX + AMP + CEF + CIP + ENR + OFL + FFC	7	1	0.64
DOX + TET + AMP + SPT + CIP + FFC + SUL	7	1	0.64

As shown in Table 4, all the 16 *Salmonella* isolates showed resistance to at least two antimicrobials, and 12 of them exhibited an MDR phenotype. Serovars Typhimurium and Enteritidis were found most often in the MDR isolates. It should be highlighted that two *Salmonella* isolates that displayed resistance to up to 5 of the antimicrobials tested were all purchased from pork in the open‐air markets.

### 3.3 Detection of antimicrobial resistance genes

The antimicrobial resistance genes (*tetA*, *tetG*, *bla*_TEM_, *bla*_CMY_, *aadA1*, *aadA2*, *qnrS*, *aac(6′)‐Ib‐cr*, *sul1*, and *sul2*) conferring resistance to five classes of antimicrobials are shown in Table 5. All but three (81.3%) isolates contained *tetA*, while none of the isolates were positive for *tetG*. Ten out of the fourteen β‐lactam‐resistant isolates carried *bla*_TEM_, and no isolates carried *bla*_CMY_. *aadA1* and *aadA2* were observed in 10 (62.5%) and 6 (37.5%) of the 16 isolates, respectively. *qnrS* was present in a single isolate (6.3%), while *aac(6′)‐Ib‐cr* was detected in 93.8% of the isolates. *sul2* was detected in all the 16 *Salmonella* isolates, while only 4 (25%) harbored *sul1*.

**TABLE 5:** Distribution of antimicrobial resistance genes among *Salmonella* from this study

Antimicrobial	Resistance genes	No. of positive isolates (%)
Tetracyclines	*tetA*	81.3 (13/16)
	*tetG*	0.0 (0/16)
β‐Lactams	*bla*_TEM_	62.5 (10/16)
	*bla*_CMY_	0.0 (0/16)
Spectinomycin	*aadA1*	62.5 (10/16)
	*aadA2*	37.5 (6/16)
Fluoroquinolones	*qnrS*	6.3 (1/16)
	*aac(6′)‐Ib‐cr*	93.8 (15/16)
Sulfonamides	*sul1*	25.0 (4/16)
	*sul2*	100.0 (16/16)

### 3.4 Relationship between antimicrobial resistance genes and antimicrobial susceptibility

The coincidence rates of the antimicrobial resistance genes and the antimicrobial resistance phenotypes of the 16 *Salmonella* isolates ranged from 26.7% to 92.3% (Table 6). Almost all isolates (12/13) carrying *tetA* or *tetB* were resistant to tetracyclines, with similar results for aminoglycosides (9/11). In addition, 4 of the 15 isolates harboring *qnrS* or *aac(6′)‐Ib‐cr* were resistance to fluoroquinolones. Eight isolates with *sul1* or *sul2* were susceptible to sulfonamides.

**TABLE 6:** Coincidence rates of antimicrobial resistance genes and antimicrobial susceptibility of *Salmonella*

Antimicrobial classes	No. of resistant isolates	No. of isolates carrying resistant genes	Coincidence rate (%)
Tetracyclines	12	13	92.3
β‐Lactams	14	10	71.4
Aminoglycosides	9	11	81.8
Fluoroquinolones	4	15	26.7
Sulfonamides	8	16	50

### 3.5 Detection of virulence genes

In the present study, different serotypes of *Salmonella* carried different virulence genes, and the same virulence genes had different effects on the pathogenicity of *Salmonella* from different sources. The presence or absence of seven virulence genes is shown in Table 7. The size of the detected target band was consistent with the expected sizes. The *mogA*, *mgtC*, and *sopB* present in SPIs and *spvB* present in the virulent plasmids were detected in all 16 isolates, and almost all the isolates were positive for *spvC* (93.8%). Of the other virulence genes, the majority of the isolates carried *sseL* with the exception of four isolates. However, *siiE* was detected in 5 of the 16 isolates tested (31.3%). Overall, all the *Salmonella* isolates showed at least five virulence genes.

**TABLE 7:** Percentage of virulence genes in *Salmonella* isolated from pork, chicken, and duck

Virulence genes	Over all (%)	Pork (%)	Chicken (%)	Duck (%)
*invA*	100.0 (16/16)	100.0 (3/3)	100.0 (10/10)	100.0 (3/3)
*mogA*	100.0 (16/16)	100.0 (3/3)	100.0 (10/10)	100.0 (3/3)
*sseL*	75.0 (12/16)	100.0 (3/3)	70.0 (7/10)	66.7 (2/3)
*mgtC*	100.0 (16/16)	100.0 (3/3)	100.0 (10/10)	100.0 (3/3)
*siiE*	31.3 (5/16)	33.3 (1/3)	30.0 (3/10)	33.3 (1/3)
*sopB*	100.0 (16/16)	100.0 (3/3)	100.0 (10/10)	100.0 (3/3)
*spvB*	100.0 (16/16)	100.0 (3/3)	100.0 (10/10)	100.0 (3/3)
*spvC*	93.8 (15/16)	100.0 (3/3)	90.0 (9/10)	100.0 (3/3)

## 4 DISCUSSION

*Salmonella* is often found in foods of meat origin and is implicated in many cases of human illness. In previous studies, the prevalence of *Salmonella* among retail meats in China varied widely (Chen et al., 2020; Yang et al., 2020; Zhang et al., 2018). In our study, the results for the proportion of *Salmonella*‐positive meat (13.3%) were similar to those reported by Sodagari et al. (2015), who found 19.8% in retail chicken meat and giblets in Iran, and Yu et al. (2014), who reported 46 positive samples from 367 raw meat samples, collected from supermarkets and open‐air markets in Henan (12.5%). This variation in the prevalence rates in different regions might be biased because of the types of meat samples, slaughterhouse sanitation, sampling season, and detection methods (Ou et al., 2020; Oueslati et al., 2016; Sallam et al., 2014).

Our study revealed that more chicken samples were positive for *Salmonella* than duck and pork samples, with no significant difference (*p* > 0.05), which was in agreement with the results reported by Yang et al. (2010) and Ou et al. (2020). This indicated that *Salmonella* contamination in retail meats in Anhui Province was serious, especially in chicken. This may be because of poor hygiene practices at the stage of product preparation and selling. In addition, the isolation rate of *Salmonella* collected from open‐air markets was remarkably higher than that from supermarket chains (*p* < 0.05), which was in concordance with the report of Zwe et al. (2018). Poor sanitary practices, such as cleaning knives and cutting boards untimely and not wearing masks and gloves could likely bring about cross‐contamination events. Regarding this issue, the supervision of open‐air markets should be strengthened to ensure high standards of environmental hygiene and reduce the risk of human infection.

Six *Salmonella* serovars were identified in the present study, dominated by *S*. Enteritidis, *S*. Typhimurium, and *S*. Typhi. Of note, all five *S*. Enteritidis isolates were present in chicken samples, and *S*. Enteritidis was also the dominant serovar from retail chicken in other survey studies (Ren et al., 2016; Yang et al., 2010), but another study from Singapore reported *S*. Saintpaul as the most common serotype in retail fresh chicken meat, followed by *S*. Brancaster (Zwe et al., 2018). A previous study by Furukawa et al. (2016) reported that *S*. Infantis was the most frequent in retail poultry meat in Japan. In Nigeria, *S*. Amoutive, the serotype isolated most frequently from retail beef, chicken, goat, and pork samples (Smith et al., 2016), was not detected in our study. The prevalence of *Salmonella* serovars in retail meats may vary greatly by geographic region, temporal changes, and different breeds of meat.

As reported in the USA (82%) (Chen et al., 2004), Singapore (80.8%) (Zwe et al., 2018), and northern India (100%) (Sharma et al., 2019), we noted a high prevalence of antimicrobial resistance of *Salmonella* isolates from retail meats. Similarly, all *Salmonella* isolates were resistant to the 11 antimicrobial agents in this study. For each antimicrobial agent, the antimicrobial with the highest resistance rate in this study was ampicillin, which was consistent with the results of reports from Wuhan (Zhu et al., 2020) and Yangzhou (Li, Yin, et al., 2019). The resistance of *Salmonella* to ampicillin has become commonplace in Africa and the USA owing to its extensive clinical use (Ke et al., 2014). Our findings also showed that the frequency of ampicillin resistance among isolates from supermarket chains was lower than that from open‐air markets. Further studies with larger sample sizes are needed to more accurately determine whether there are differences in antimicrobial resistance between *Salmonella* isolates from different locations.

Notably, resistance was frequently observed in tetracyclines, and active efflux systems are one of the main mechanisms of tetracyclines resistance. Our results were comparable to those of Yang et al. (2019), in which 65.6% of *Salmonella* isolates from retail meat and meat products in China were resistant to tetracycline. In northern Vietnam, resistance to tetracycline was also commonly observed in isolates from beef in retail markets (Thai et al., 2012). However, low‐level tetracycline resistance in *Salmonella* has previously been reported in most developed countries and regions (Kim et al., 2012; Nisar et al., 2017). These results could be related to the unsuitable or unrestricted use of corresponding antimicrobials in livestock farming, particularly in developing countries (Chen et al., 2020).

A relatively high resistance rate to enrofloxacin and ciprofloxacin was observed in this study. Fortunately, only one isolate was resistant to ofloxacin, whereas fluoroquinolone‐resistant isolates have emerged and are at high levels of resistance (Ou et al., 2020). These isolates should be of great concern, as second‐generation cephalosporins and third‐generation quinolones are the main antimicrobials of choice for *Salmonella* infections (Yang et al., 2016). A report by Smith et al. (2016) in 2013 from Lagos, Nigeria, stated that all of the tested isolates were susceptible to ofloxacin and ciprofloxacin; however, in the present study, 18.8% of the isolates were resistant to enrofloxacin, 12.5% were resistant to ciprofloxacin, and 6.3% were resistant to ofloxacin.

Our results of antimicrobial resistance of *Salmonella* were similar to those from the study by Zhang et al. (2018), who assayed for antimicrobial susceptibility of 615 *Salmonella* isolates obtained from chicken and pork in retail markets in Guangdong, China. The study showed that resistance to tetracycline, gentamicin, ciprofloxacin, florfenicol, and sulfisoxazole reached 75.3%, 16.3%, 12%, 38.7%, and 76.1%, respectively. However, in Canada, the percentage of resistance to ciprofloxacin, ampicillin, gentamicin, sulfisoxazole, and tetracycline was lower than in our study, except for ceftiofur (Aslam et al., 2012), which may indicate a better control of such antimicrobials in retail meats in Canada.

By analyzing total antibiotic resistance profiles, 12 isolates tested were resistant to three or more antimicrobial agents, accounting for 75% of the total number of isolates, which caused difficulties in the clinical treatment of *Salmonella*. The spread of MDR isolates was potentially severe: 60.7% in Korea (Seo et al., 2019), 88.1% in southern China (Chen et al., 2020), and 100% in Egypt (Sallam et al., 2014). The high level of antimicrobial resistance was directly related to the unscientific use of broad‐spectrum and cheap antimicrobials in the rearing of livestock and poultry, resulting in an increasing number of antimicrobial‐resistant and MDR bacteria. It is noteworthy that two isolates obtained from open‐air markets were resistant to seven different antimicrobials. The spread of MDR animal bacteria to humans threatens human health worldwide and could lead to some diseases with no remedy. Thus, it is necessary to strengthen the monitoring of the trends in the resistance spectrum of *Salmonella* to control MDR isolates.

The results of the detection of antimicrobial resistance genes showed that all five classes of antimicrobial resistance genes were detected at different degrees, among which the superior resistance genes were *tetA* (81.3%), *bla*_TEM_ (62.5%), *aadA1* (62.5%), *aac(6′)‐Ib‐cr* (93.8%), and *sul2* (100.0%) (Table 5). The results showed that the coincidence rate of antimicrobial‐resistant phenotypes and antimicrobial‐resistant genes of the five classes of antimicrobials was over 60%. The coincidence rate of sulfonamides was the highest, up to 100.0%. *sul2*, located on non‐conjugative plasmids or a transmissible multi‐resistance plasmid (Nghiem et al., 2017), was the main sulfonamide resistance gene, which was consistent with the report by Zhu et al. (2017). However, Chen et al. (2020) found that *sul1* was the main sulfonamide resistance gene detected in *Salmonella* recovered from retail duck meat in Southern China. This may be because of the different types of *Salmonella* among various regions, resulting in distinct resistance genes.

Recently, fluoroquinolones have been the most commonly used antimicrobials in the clinical control of *Salmonella* infection. Plasmid‐mediated quinolone resistance (PMQR) has emerged in *Salmonella* spp. and in other *Enterobacteriaceae* with increasing prevalence (Pribul et al., 2016). PMQR determinants generally generate only low‐level resistance, but nonetheless can complement the mechanisms of chromosomal resistance to reach clinical resistance levels (Wang et al., 2020). The recent discovery of PMQR could result in horizontal transfer of fluoroquinolone resistance between strains (Hopkins et al., 2005). *qnrS* mediates low levels of fluoroquinolone resistance by altering the role of the antimicrobial target site and then binding to the target enzyme, while the *cr* variant of *aac(6′)‐Ib* encodes an aminoglycoside acetyltransferase that confers reduced susceptibility to ciprofloxacin by *N*‐acetylation of its piperazinyl amine. *aac(6′)‐Ib‐cr* has two amino acid changes, Trp102Arg and Asp179Try, which together are necessary and sufficient for the enzyme's ability to acetylate ciprofloxacin (Park et al., 2006). The results of this study revealed that most isolates carried *aac(6′)‐Ib‐cr* (93.8%), which was similar (79.17%) to the data reported previously in Changchun (Ren et al., 2016). However, the detection rate of *aac(6′)‐Ib‐cr* was relatively low in Tunisia (1.7%) and Brazil (17.8%), depending on the location (Al‐Gallas et al., 2013). Since PMQR is probably the primary mechanism of quinolone resistance in animal‐derived foods, we conjecture that it was also the cause of fluoroquinolone resistance in this study.

Tetracycline resistance was mainly encoded by *tetA*, and in our study, a high prevalence of *tetA* was detected in the examined isolates. The superiority of *tetA* in *Salmonella* played an outstanding role in tetracycline resistance. However, no isolates were found to harbor *tetG*, and a similar phenomenon that did not find *tetG* among the three genes screened (*tetA*, *tetB*, *tetG*) has been suggested previously in Northern India (Sharma et al., 2019).

In Gram‐negative bacteria, resistance β‐lactams is mediated by different strategies such as production of β‐lactamases, efflux pumps, and alteration of penicillin binding proteins (Ma et al., 2017). The results of the detection of β‐lactam resistance genes showed that *bla*_TEM_ was the most frequent gene; however, *bla*_CMY_ was not detected in any *Salmonella* isolates. The β‐lactam resistance genes were of concern because the utilization of β‐lactam antimicrobials among patients reached 70% in China (Ren et al., 2016). Fortunately, *bla*_CMY_‐positive *Salmonella* has been found in China at a low rate and has only been reported in Shanxi and Sichuan (Zhao et al., 2017).

The data on antimicrobial resistance genes and phenotypes indicate that the *Salmonella* isolates had a high correlation between the phenotypes and genotypes of tetracyclines, β‐lactams, and aminoglycosides, while the coincidence rate of phenotypes and genotypes of fluoroquinolones and sulfonamides was relatively lower. This discrepancy indicates that the resistance phenotype was associated with the existence of resistance genes, but it did not rigidly follow a one‐to‐one correspondence, which was in connection with the complex mechanisms of antimicrobial resistance of *Salmonella* isolates (Chen et al., 2020). Factors, such as the expression of resistance genes, the interaction between different resistance genes, gene mutation, and biofilm formation affected the resistance to antimicrobials, or the resistance phenotypes encoded by the detected resistance genes did not match with the antimicrobials in this study, which resulted in the deviation between the genotypes and the resistance phenotypes (Ren et al., 2016). Comparisons of sequencing results of antimicrobial resistance genes showed that the sequences of antimicrobial resistance genes were remarkably similar to the reference sequences, which were all above 99%.

Pathogenicity in *Salmonella* is complex and multifactorial (Carvalho et al., 2017). Bacterial infections of the host usually adhere to the host cell, and then successfully invade the host cell through its virulence factors, resulting in the disease and even death of the host (Tang & Holden, 1999). This is due to the presence of the genetic determinants of virulence in *Salmonella*. High frequencies were reported for *mogA* (100%), *mgtC* (100%), *sopB* (100%), *spvB* (100%), and *spvC* (93.8%), with the exception of *sseL* (75.0%) and *siiE* (31.3%), which provided further evidence that these virulence genes were widespread in *Salmonella*. Based on virulence profiles, more than 68.8% of isolates harbored over six virulence genes, and 31.3% were positive for all the virulence genes tested. The more virulence genes the isolates harbored, the more likely they were to show pathogenicity and the stronger the virulence. Several studies have shown that the virulence and pathogenicity of *Salmonella* decrease when *sseL* (SPI‐2) is mutated, and pathogenicity is mainly related to SPI‐1 and SPI‐2 (Ochman et al., 1996). In this study, we found a high prevalence of *spvB* and *spvC* in *Salmonella*. Virulence plasmid genes are conducive to the adhesion and settlement of bacteria in host cells and improve the viability of host cells, which may be the reason why virulence plasmid genes enhance the pathogenicity of *Salmonella* (Haneda et al., 2001). In general, the high detection rate of virulence genes highlighted the pathogenic potential of these isolates, playing an important role in human disease.

## 5 CONCLUSION

In conclusion, the present study investigated the occurrence, antimicrobial resistance, antimicrobial resistance genes, and virulence genes of *Salmonella* among retail meats in Anhui, which demonstrated that retail meats, especially chicken, showed a high incidence of *Salmonella* exhibiting MDR phenotypes. The high rate of *sul2* and *aac(6′)‐Ib*‐cr‐positive isolates suggested that the *Salmonella* surveillance system and the reasonable use of antimicrobials in livestock and poultry production would help combat and control *Salmonella* infection. Moreover, the genes *mogA*, *mgtC*, *sopB*, and *spvB* were found in all the *Salmonella* isolates. Further studies with large numbers of *Salmonella* isolates along the food chain are the required need to explore the epidemiological situation and to track and prevent salmonellosis.

## CONFLICT OF INTEREST

The authors declare no conflicts of interest.

## Supporting information

Supplementary Material
