# Supplementary material for: Occurrence and antimicrobial resistance of Salmonella isolated from retail meats in Anhui, China
Source: Food Sci Nutr. 2021 Jul 16;9(9):4701–10. doi: 10.1002/fsn3.2266 (PMC8441314; doi:10.1002/fsn3.2266)
Supplement: Supplementary file 1 — Supplementary Material [file FSN3-9-4701-s001.docx]

**Table S1** Primer sequences and relevant information of virulence genes used in the current study.

| Genes | Primer sequence (5' to 3') | Size (bp) | Tm (°C) | Reference |  |  |  |
| --- | --- | --- | --- | --- | --- | --- | --- |
| *tetA* | F: GCTACATCCTGCTTGCCTTC | 210 | 58 | (Carlson et al., 1999) | | | |
|  | R: CATAGATCGCCGTGAAGAGG |  |  |  |  |  |  |
| *tetG* | F: GCTCGGTGGTATCTCTGCTC | 468 | 59 | (Carlson et al., 1999) | | | |
|  | R: AGCAACAGAATCGGGAACAC |  |  |  |  |  |  |
| *bla*_TEM_ | F: GGTCCTCCGATCGTTGTCAG | 310 | 68 | (Carlson et al., 1999) | | | |
|  | R: TTCATCCATAGTTGCCTGACT |  |  |  |  |  |  |
| *bla*_CMY_ | F: ACAGAACAACAGATTGCCGATA | 856 | 54 | (Li et al., 2013) | | | |
|  | R: TGTCGCTGCCGTTGATGA |  |  |  |  |  |  |
| *aadA1* | F: TTTGCTGGTTACGGTGAC | 497 | 56 | (Zhu et al., 2019) | | | |
|  | R: GCTCCATTGCCCAGTCG |  |  |  |  |  |  |
| *aadA2* | F: TGTTGGTTACTGTGGCCGTA | 381 | 68 | (Ng et al., 1999) | | | |
|  | R: GCTGCGAGTTCCATAGCTTC |  |  |  |  |  |  |
| *qnrS* | F: ACGACATTCGTCAACTGCAA | 417 | 53 | (Pribul et al., 2016) | | | |
|  | R: TAAATTGGCACCCTGTAGGC |  |  |  |  |  |  |
| *aac(6')-Ib* | F: TTGCGATGCTCTATGAGTGGCTA | 482 | 62 | (Park et al., 2006) | | | |
|  | R: CTCGAATGCCTGGCGTGTTT |  |  |  |  |  |  |
| *sul1* | F: TGCAGGCTGGTGGTGGTTA | 425 | 58 | (Zhu et al., 2019) | | | |
|  | R: CGCGTGGGTGCGGACGT |  |  |  |  |  |  |
| *sul2* | F: CATTCCCGTCTCGCTCGA | 435 | 52 | (Zhu et al., 2019) | | | |
|  | R: GCGCGCAGAAAGGATTT |  |  |  |  |  |  |
| *invA* | F: GTGAAATTATCGCCACGTTCGGGCAA | 284 | 62 | (Bülte & Jakob, 1995) | |  |  |
|  | R: TCATCGCACCGTCAAAGGAACC |  |  |  |  |  |  |

Table S1 (continued)

| Genes | Primer sequence (5'to 3') | Size (bp) | Tm (°C) | | Reference | |  |  |  |
| --- | --- | --- | --- | --- | --- | --- | --- | --- | --- |
| *mogA* | F: ATTGGCTTAGTTTCTATCTCCG | 200 | 56 | | (Li et al., 2019) | | | |  |
|  | R: CCTTCCAGCGTTTCTTTGA |  |  | |  | |  |  |  |
| *sseL* | F: GCCCCTTCCAGATTACTTTATATG | 269 | 57 | | (Hai et al., 2020) | | | | |
|  | R: TGCTTAATATATTTTCTTTGGTGG |  |  | |  | |  |  |  |
| *mgtC* | F: AAAGACAATGGCGTCAACGTATGG | 500 | 65 | | (Hai et al., 2020) | | | | |
|  | R: TTCTTTATAGCCCTGTTCCTGAGC |  |  | |  | |  |  |  |
| *siiE* | F: TTTTTTGCCGATCAAAATTCTGTA | 750 | 54 | | (Hai et al., 2020) | | | | |
|  | R: TATACTATCATCTTTGCTACCGCT |  |  | |  | |  |  |  |
| *sopB* | F: TCAGAACTCGTCTAACCACTC | 517 | 58 | | (Hai et al., 2020) | | | | |
|  | R: TACCGTCCTCATGCACACTC |  |  | |  | |  |  |  |
| *spvB* | F: CCTGATGTTCCACCACTTTC | 590 | 60 | | (Hai et al., 2020) | | | | |
|  | R: ATGCCTTATCTGGCGATGT |  |  | |  | |  |  |  |
| *spvC* | F: AAGGTCGTTCAACAAGCC | 252 | 54 | | (Hai et al., 2020) | | | | |
|  | R: CATTTCACCACCATCACG |  | |  | |  |  |  |  |

**References**

Bülte, M., & Jakob, P. (1995). The use of a PCR-generated *invA* probe for the detection of *Salmonella* spp. in artificially and naturally contaminated foods. *International Journal of Food Microbiology, 26*(3), 335-344. DOI: 10.1016/0168-1605(94)00139-W

Carlson, S. A., Bolton, L. F., Briggs, C. E., Hurd, H. S., Sharma, V. K., Fedorka-Cray, P. J., & Jones, B. D. (1999). Detection of multiresistant *Salmonella Typhimurium* DT104 using multiplex and fluorogenic PCR. *Molecular and Cellular Probes, 13*(3), 213-222. DOI: 10.1006/mcpr.1999.0240

Hai, D., Yin, X., Lu, Z., Lv, F., Zhao, H., & Bie, X. (2020). Occurrence, drug resistance, and virulence genes of *Salmonella* isolated from chicken and eggs. *Food Control, 113,* 107109. DOI: 10.1016/j.foodcont.2020.107109

Li, R., Lai, J., Wang, Y., Liu, S., Li, Y., Liu, K., Shen, J., & Wu, C. (2013). Prevalence and characterization of *Salmonella* species isolated from pigs, ducks and chickens in Sichuan Province, China. *International Journal of Food Microbiology, 163*(1), 14-18. DOI: 10.1016/j.ijfoodmicro.2013.01.020

Li, Q., Yin, J., Li, Z., Li, Z., Du, Y., Guo, W., Bellefleur, M., Wang, S., & Shi, H. (2019). Serotype distribution, antimicrobial susceptibility, antimicrobial resistance genes and virulence genes of *Salmonella* isolated from a pig slaughterhouse in Yangzhou, China. *AMB Express, 9*(1), 210. DOI: 10.1186/s13568-019-0936-9

Ng, L. K., Mulvey, M. R., Martin, I., Peters, G. A., & Johnson, W. (1999). Genetic characterization of antimicrobial resistance in Canadian isolates of *Salmonella* serovar Typhimurium DT104. *Antimicrobial Agents & Chemotherapy, 43*(12), 3018-3021. DOI: 10.1128/AAC.43.12.3018

Park, C. H., Robicsek, A., Jacoby, G. A., Sahm, D., & Hooper, D. C. (2006). Prevalence in the United States of *aac(6′)-Ib-cr* encoding a ciprofloxacin-modifying enzyme. *Antimicrobial Agents and Chemotherapy, 50*(11), 3953-3955. DOI: 10.1128/AAC.00915-06

Pribul, B. R., Festivo, M. L., Souza, M. M. S. D., & Rodrigues, D. D. P. (2016). Characterization of quinolone resistance in *Salmonella* spp. isolates from food products and human samples in Brazil. *Brazilian Journal of Microbiology, 47*(1), 196-201. DOI: 10.1016/j.bjm.2015.04.001

Zhu, A., Zhi, W., Qiu, Y., Wei, L., Tian, J., Pan, Z., Kang, X., Gu, W., & Duan, L. (2019). Surveillance study of the prevalence and antimicrobial resistance of *Salmonella* in pork from open markets in Xuzhou, China. *Food Control, 98*, 474-480. DOI: 10.1016/j.foodcont.2018.07.035
